# Characterization of TET and IDH gene expression in chronic lymphocytic leukemia: comparison with normal B cells and prognostic significance

**DOI:** 10.1186/s13148-016-0298-y

**Published:** 2016-12-07

**Authors:** Michaël Van Damme, Emerence Crompot, Nathalie Meuleman, Marie Maerevoet, Philippe Mineur, Dominique Bron, Laurence Lagneaux, Basile Stamatopoulos

**Affiliations:** 1 Laboratory of Clinical Cell Therapy, ULB Cancer Research Center (U-CRC), Institut Jules Bordet, Université Libre de Bruxelles (ULB), Route de Lennik, 808, 1070 Brussels, Belgium; 2 Department of Hematology (U-CRC), Institut Jules Bordet, Université Libre de Bruxelles (ULB), Brussels, Belgium; 3 Department of Hemato-Oncology, Grand Hôpital de Charleroi, Gilly, Belgium

**Keywords:** Chronic lymphocytic leukemia, TET, IDH, 5-Hydroxymethylcytosine, Prognosis

## Abstract

**Background:**

Chronic lymphocytic leukemia (CLL) is the most common hematological malignancy in western countries, characterized by a heterogeneous clinical course. Although genetic studies have identified chromosomal aberrations or specific mutations, epigenetic changes have been poorly characterized in CLL.

**Methods:**

We assessed ten-eleven translocations (TET) 1, 2, and 3, isocitrate dehydrogenase (IDH) 1, and 2 messenger RNA (mRNA) expression using real-time PCR on purified leukemic B cells from 214 CLL patients (median follow-up = 75 months, range 1–380), normal peripheral blood B cells (*n* = 20), and umbilical cord blood B cells (*n* = 21). The microenvironment influence was assessed after 24 h co-culture of CLL cells with bone marrow mesenchymal stromal cells (BMSC). Finally, 5-hydroxymethylcytosine level (%5-hmC) was assessed by ELISA in CLL cells alone or with microenvironment stimuli.

**Results:**

TET 1 and 3 and IDH2 were decreased in CLL cells compared with healthy B cells (*P* = 0.0221, 0.0013, <0.0001, respectively), while IDH1 was overexpressed (*P* = 0.0037). TET2 and IDH1 were significantly correlated with treatment-free survival (TFS); patients with high TET2/IDH1 expression had a higher median TFS (111 months) than patients with low expression (78 months, *P* = 0.0071/0.0123). Moreover, TET1 expression decreased (*P* = 0.0371), while TET3 and IDH2 expression increased (*P* = 0.0273/0.0039) in co-cultures. However, %5-hmC was not correlated with clinical data and was unchanged following microenvironment stimuli.

**Conclusions:**

Despite a slight deregulation in CLL cells compared with normal B cells, we identified a significant association between TET/IDH gene expression and prognosis, suggesting that epigenetic changes could potentially be associated with disease progression. Moreover, despite an identical %5-hmC, TET gene expression was influenced by contact with BMSC confirming the crucial role of the microenvironment in CLL pathogenesis.

**Electronic supplementary material:**

The online version of this article (doi:10.1186/s13148-016-0298-y) contains supplementary material, which is available to authorized users.

## Background

Chronic lymphocytic leukemia (CLL) is the most common hematological malignancy in the west and is characterized by a heterogeneous clinical course [1]; some patients will live several decades without any symptoms, while others will rapidly require a treatment and will have a decreased overall survival (OS). Clinical and molecular factors, such as Binet stage, lymphocyte doubling time (LDT), mutational status of the immunoglobulin heavy-chain variable-region (IgHV), zeta-chain-associated protein kinase 70 (ZAP70), lipoprotein lipase (LPL) or CD38 expression, and serum levels of soluble CD23 (sCD23) and beta-2-microglobulin (B2M), can be used to classify patients into different prognostic subgroups [2]. Moreover, increasing evidence suggests a role for the microenvironment in CLL pathogenesis. Our group previously demonstrated that bone marrow mesenchymal stromal cells (BMSC) protect CLL but not normal B cells from apoptosis through direct contact [3].

While genetic lesions, such as chromosomal aberrations [4] or specific mutations, [5–8] are involved in CLL physiopathology, in recent years, growing evidence has suggested that epigenetic characteristics are key factors in leukemic processes. Recent studies have investigated epigenetic features and demonstrated the importance of DNA methylation [9] or histone post-translational modifications in prognosis, oncogene regulation, or therapeutic targeting [10–12]. We demonstrated in previous papers that histone deacetylase (HDAC) mRNA expression was associated with poor (HDAC7, HDAC10, and SIRT5) or good prognosis (HDAC6, SIRT3, and SIRT6) [13] in CLL patients. Moreover, global HDAC enzymatic activity is a strong predicator of poor prognosis in CLL which can refine well-known prognostic factors [14].

In 2009, Tahiliani and colleagues discovered 5-hydroxymethylcytosine (5-hmC) as the sixth base of the DNA in mammalian cells [15]. Ten-eleven translocation proteins (TET) are the dioxygenases responsible for the oxidation of 5-methylcytosine (5-mC) to form 5-hmC. There are three known TET isoenzymes (TET1, 2, and 3), and they require oxygen, Fe(II), and 2-oxoglutarate for their activity. This last cofactor is produced in the Krebs cycle by the isocitrate dehydrogenases (IDH) 1 and 2. Other subsequent studies suggested that the 5-hmC marker could be a step in the demethylation process [16–21] and/or a pattern allowing specific enzymes to bind hydroxymethylated regions of the genome [22–26].

Hydroxymethylation enzyme defects have previously been associated with hematological malignancies; mutations in TET2 were found in acute myeloid leukemia (AML) or chronic myelomonocytic leukemia (CMML) and induced loss of hydroxymethylation and were linked with poor prognosis [27–30]. However, reports on TET2 mutations in B cell neoplasms are rare [31], and little is known about DNA hydroxymethylation in CLL.

In the present study, we measured the mRNA expression of TET1, 2, and 3 and IDH1 and 2 by quantitative real-time PCR (qPCR) in highly purified CD19+ B cells from a large cohort of CLL patients (*n* = 214) and correlated these data with clinical outcome. We also investigated the potential association between the global DNA 5-hmC rate and microenvironment stimuli, especially BMSC.

## Methods

### Patients and samples

This study was approved by the Bordet Institute Ethics Committee and conducted according to the principles expressed in the Declaration of Helsinki. All samples were collected at the time of diagnosis before any treatment, and after, written informed consent was obtained from 214 CLL patients who presented with a typical CD19+CD5+CD23+ phenotype and a Catovsky score of 4 or 5/5. Treatment-free survival (TFS) and OS were calculated from the time of diagnosis until the date of first treatment and the date of death, respectively. All deaths were CLL-related. Control samples were obtained from the peripheral blood of 20 age-matched healthy volunteers (PBHV) (mean 69 years old, range 54–90) after written informed consent was obtained and from 21 umbilical cord blood (UCB) samples after full-term delivery and written informed consent of the mothers was obtained. Leukemic B cells were isolated from mononuclear cells with magnetic beads targeting CD19+ phenotype (MidiMACS, Miltenyi Biotec). Briefly, pellet of 10 million mononuclear cells were resuspended in 80 μl of PBS-05% BSA and 20 μl of CD19 microbeads. After 15 min of incubation at 4 °C and washing, cell suspension was applied onto a column placed in a magnetic field. Three washes were performed to discard unlabeled cells and then the column was removed to the separator and placed on a collector tube where magnetically labeled cells were flushed out. For experiments which require culture, cells were negatively isolated by an indirect magnetic labeling system (B-CLL Cell Isolation Kit from MidiMACS, Miltenyi Biotec) to avail any cell activation or apoptosis. The isolation process is performed in two steps: a first labeling with a cocktail of biotin-conjugated monoclonal anti-human antibodies and then, after incubation, an adding of anti-biotin microbeads. CD19+ cells are directly collected during the elution of the column.

### BMSC isolation and conditioned medium preparation

BMSC were harvested from the sternum or iliac crest of healthy volunteers and were isolated by the classical adhesion method, as previously described [32]. Conditioned media were prepared from 24 h cultures of BMSC alone or CLL B cells + BMSC with and without contact (separated by a 0.4-μm pore-size filter). Cultures of CLL B cells with BMSCs or different conditioned media were performed for 24 h.

### Assessment of classic prognostic factors

ZAP70 and LPL were measured by qPCR as previously described [33]. CD38 expression was assessed by flow cytometry, sCD23 and B2M were measured by ELISA, and IgHV gene mutational analysis was performed using the IGH Somatic Hypermutation Assay v2.0 (Invivoscribe–Ref. 5-101-0031). LDT was assessed according to Montserrat et al. [34]. Classical cytogenetics by standard karyotype analysis and additional interphase FISH were performed to screen for the most common aberrations using the Chromoprobe Multiprobe® CLL System (Cytocell, Amplitech–Ref. PMP 018/017/016/020). Patients were then classified according to the recommendations of Döhner and Cuneo et al. [4, 35]. Additional details can be found in Additional file 1: Text 1. All of these factors, except B2M for OS, were shown to be significant predictors of TFS and OS, indicating that our cohort is representative of a CLL population (Additional file 1: Figure S1 and Table S1).

### RNA and DNA extraction and expression quantification

Total RNA was extracted from purified CD19+ cells in a single step using TriPure Isolation Reagent (Roche Life Science–Ref. 11 667 165 001). Complementary DNA (cDNA) was generated from 500 ng of RNA using qScript cDNA SuperMix (Quanta Biosciences–Ref. 95048-100) according to the manufacturer’s protocol. TET1, 2, and 3 and IDH1 and 2 mRNA expression was quantified by real-time PCR using SYBR Green technology (Applied Biosystems–Ref. 4367659). Gene expression was normalized to the cyclophilin A (PPIA) gene as an endogenous control as previously described [13] and calibrated by subtracting 10 (chosen arbitrarily) from the ΔCt. The comparative ΔΔCt method was then used for data analysis, and the fold changes were subsequently calculated (fold change = 2^−ΔΔCt^). All primer sequences are available in the Additional file 1: Text 1 (Life Technologies). Genomic DNA (gDNA) was extracted from purified CD19+ cells with a QIAamp DNA Blood Mini Kit (Qiagen–Ref. 51104).

### Assessment of 5-hmC levels

We measured the 5-hydroxymethylcytosine percentage (%5-hmC) using a “Quest 5-hmC DNA ELISA Kit” (Zymo Research–Ref. D5425). Briefly, 150 ng of denatured DNA was added to wells coated with 200 ng of anti-5-hydroxymethylcytosine polyclonal antibodies. The signal was detected at 405 nm after washing and adding the anti-DNA HRP (horseradish peroxidase) antibodies. Optic density was converted to %5-hmC using a standard curve based on five controls (with values of 0, 0.03, 0.12, 0.23, and 0.55%) provided in the kit. To quantify %5-hmC, we normalized the ELISA results with data obtained from the quantification of three genes (glyceraldehyde-3-phosphate dehydrogenase (GAPDH), actin, and PPIA) in each gDNA sample. Because these three endogenous controls produced similar results (indicating that an equivalent DNA quantity was loaded), we only used GAPDH and expressed the results as %5-hmC/GAPDH.

### Statistical analysis

The patients were stratified according to low and high TET or IDH expression with a cut-off value set by recursive partitioning maximizing the concordance with TFS as previously described for other prognostic factors [14]. All median comparisons were performed with a Mann–Whitney test or a Wilcoxon test for paired comparisons. TFS and OS analyses were performed with the Kaplan–Meier curves, and differences between groups in terms of prognosis were assessed with a log-rank test. All tests were two-sided. Differences were considered statistically significant at *P* < 0.05. All analyses were performed using the GraphPad Prism 5.0 (GraphPad Software) or SPSS 18.0.0 software.

## Results

### Population

The median age of the population was 63 years (range 34–86). The median treatment-free survival (TFS) was 88.07 months (range 0.33–251.43), and the median OS was 241.87 months (range 0.40–380.20). The median follow-up was 74.53 months (range 0.40–380.20). In this population, prognostic factors, such as IgHV mutational status, ZAP70, CD38, Binet stage, sCD23, B2M, LDT, and cytogenetic profile (cytog. profile), were significantly correlated with TFS and OS (*P* < 0.05), except B2M for OS (Additional file 1: Figure S1). Complete prognostic data were not available for all patients due to a lack of biological material. ZAP70, LPL expression, and CD38 expression and clinical data were available for all 214 patients included in this study. Incomplete data were available for IgHV mutational status (97.20%), LDT (84.11%), B2M (87.38%), sCD23 (81.78%), and cytog. profile (74.30%). All prognostic factors (except B2M for OS) were shown to be associated with prognosis, indicating that these 214 CLL patients comprise a classical CLL cohort.

### TET1 and 3 and IDH1 and 2 are deregulated in CLL

**Fig. 1:**
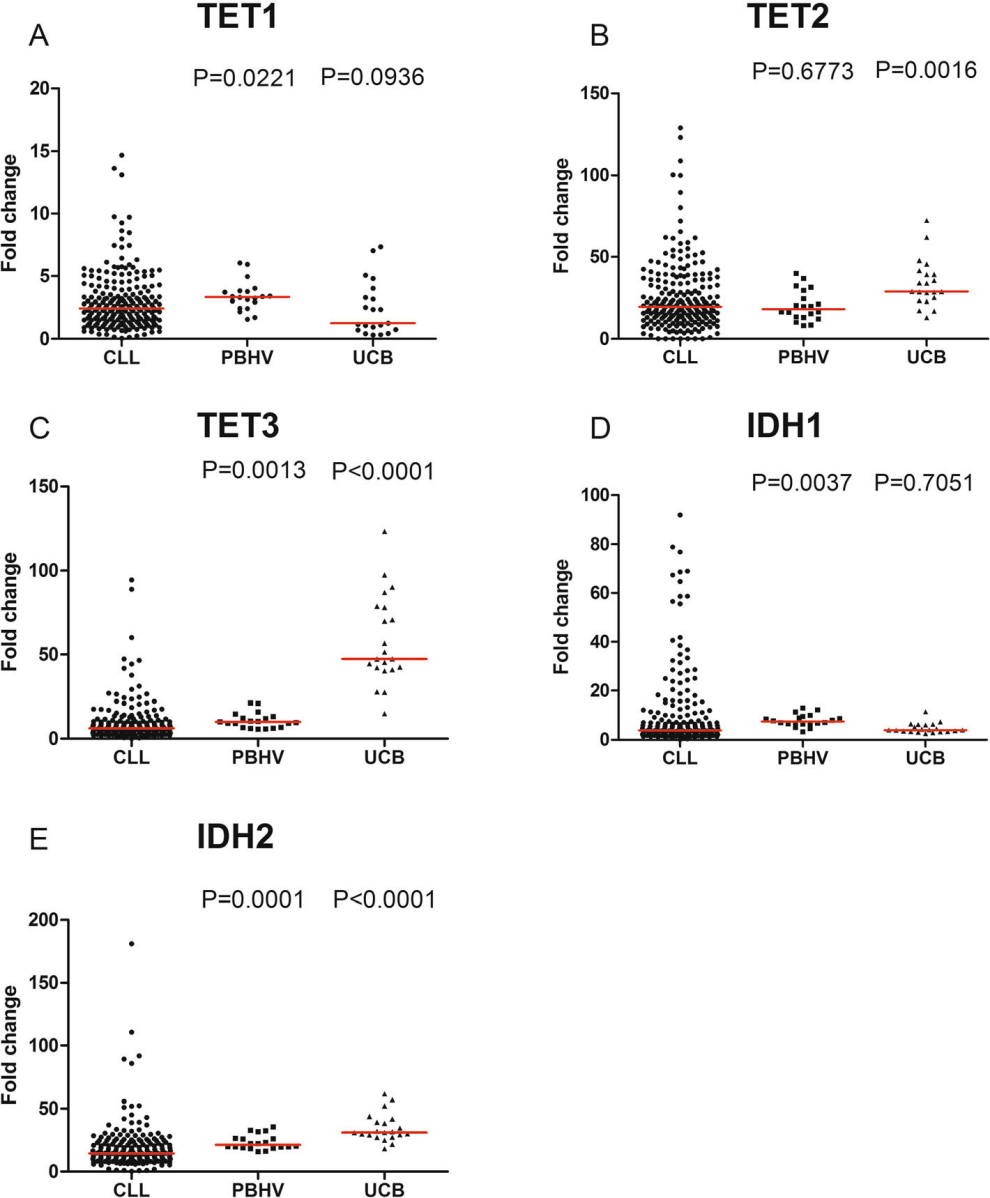
TET and IDH mRNA expression in CLL and control samples. Fold changes in mRNA expression of **a** TET1, **b** TET2, **c** TET3, **d** IDH1, and **e** IDH2 in CD19 purified cells from 214 CLL, 20 peripheral blood of healthy patients (PBHV) and 21 umbilical cord blood (UCB) samples are displayed with median (*red*). Statistical differences are indicated in relation to CLL and were assessed using the Mann–Whitney non-parametric test

TET1, 2, and 3 and IDH1 and IDH2 were measured in CD19+ CLL samples (mean purity 99.2%, range 97.5–99.9) and compared with the normal CD19+ cells. We used two types of normal counterparts: CD19+ purified B cells from the peripheral blood of healthy volunteers (mean purity 97.9%, range 95.7–100) and CD19+ purified B cells from umbilical cord blood (mean purity 98.6, range 96.4–99.5), which are CD5+ (mean 88.6% ±1.5, range 80.9–97.6), similar to CLL B cells (mean 98.0% ±0.2, range 91–100) as discussed previously. Compared with B cells from the PBHV, we observed that leukemic cells had decreased expression of TET1 and 3 and IDH2 (*P* = 0.0221, 0.0013, <0.0001, respectively) but overexpressed IDH1 (*P* = 0.0037). Compared with B cells from UCB, TET2, TET3, and IDH2 were underexpressed in leukemic cells (*P* = 0.0016, <0.0001, <0.0001, respectively) (Fig. 1). ΔCt representation of these data is also provided in Additional file 1: Figure S2. These results indicated that hydroxymethylation enzyme expression is deregulated in CLL.

### High TET2 or IDH1 expression is associated with good prognosis in terms of TFS

**Fig. 2:**
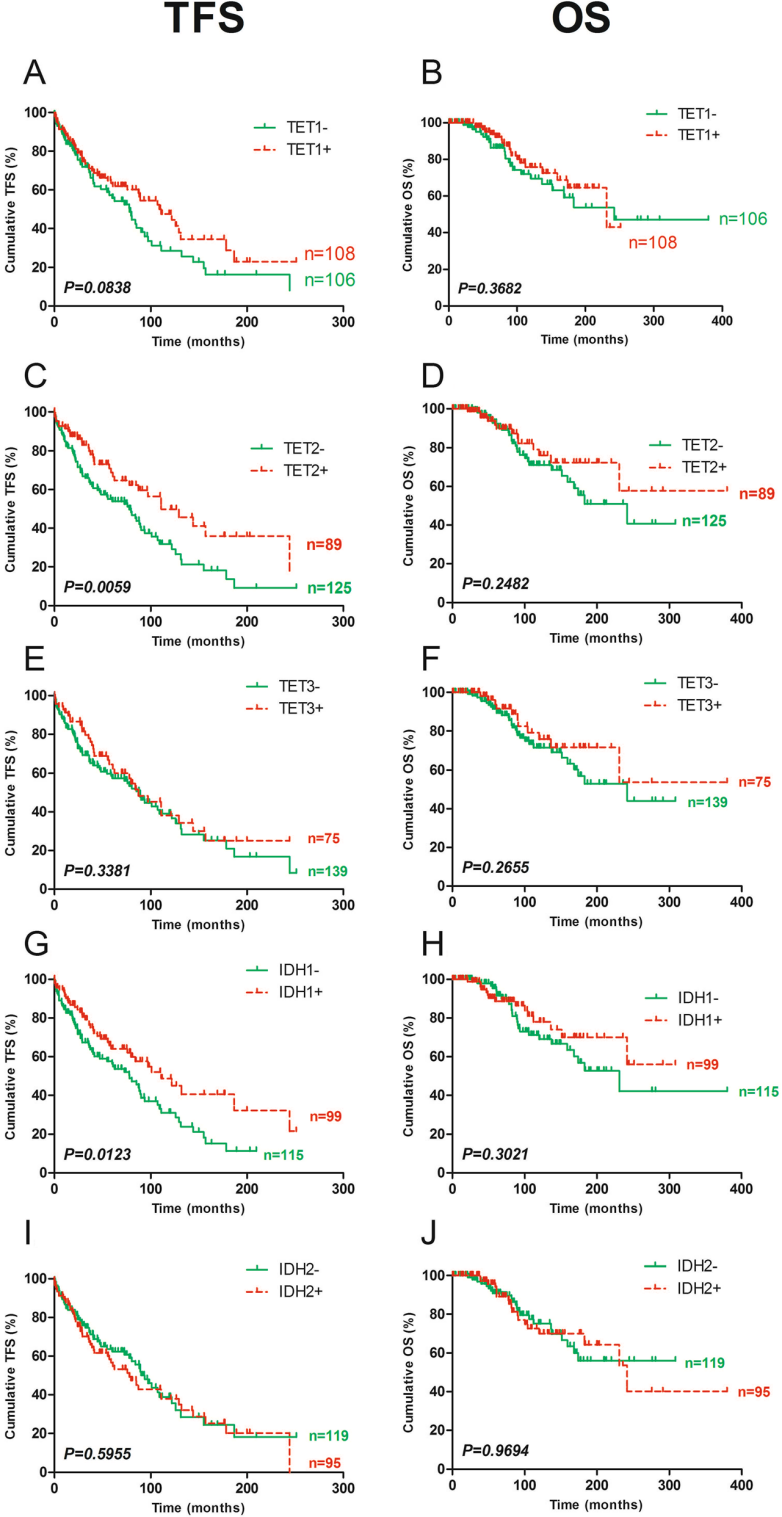
Prognostic power of TET and IDH gene mRNA expression. Fold changes in enzyme mRNA expression were measured by qPCR, and cut-offs were set by recursive partitioning. TFS and OS Kaplan–Meier curves are displayed for **a, b** TET1, **c**, **d** TET2, **e**, **f** TET3, **g**, **h** IDH1 and, **i**, **j** IDH2. Statistical differences between the curves were calculated using the log-rank test

The patients were then stratified for each studied enzyme in high and low expression groups with a cut-off value set by recursive partitioning maximizing the concordance with TFS. The cut-offs of the fold changes values for TET1, TET2, TET3, IDH1 and IDH2 were, respectively, 2.403, 22.317, 7.964, 4.268, and 15.270. TET2 and IDH1 were both significantly correlated with TFS (*P* = 0.0059 and 0.0123, respectively). Patients with high mRNA expression of TET2 or IDH1 had a median TFS of 111 months, while patients with low expression had a median TFS of 78 months. However, there were no significant correlations with OS (Fig. 2). TET and IDH gene expression was also compared between the groups based on well-known prognostic factors (IgHV mutational status, ZAP70, CD38, Binet stage, sCD23, B2M, LDT, and cytogenetic profile). No obvious and important significant differences were observed (Additional file 1: Tables S2 and S3).

### TET1, TE3, and IDH2 expression is influenced by microenvironment stimuli but global %5-hmC is unchanged

**Fig. 3:**
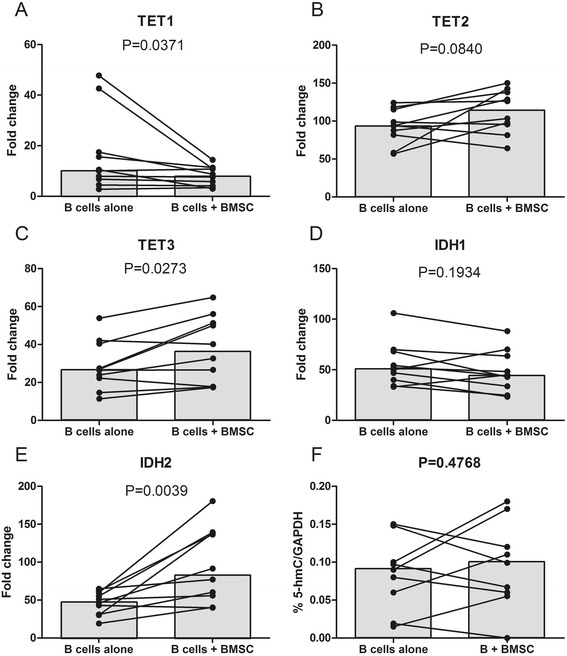
CLL B cells and BMSC co-cultures impact on TET/IDH mRNA expression and %5-hmC. Fold changes in enzyme mRNA expression of **a** TET1, **b** TET2, **c** TET3, **d** IDH1, and **e** IDH2 were plotted for ten CLL patients for samples cultured alone or with BMSC. **f ** The %5-hmC was measured by ELISA in nine CLL B cell samples cultured alone and after contact with BMSC. Significance was assessed using the Wilcoxon signed rank test

**Fig. 4:**
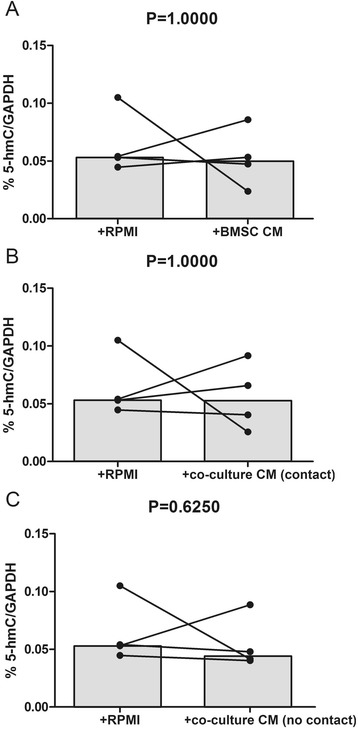
Normalized 5-hmC levels in CLL B cells cultured alone in RPMI or with conditioned medium (CM). The different conditioned media were obtained from supernatants of 24 h cultures of **a** BMSC, **b** CLL B cells + BMSC with contact, and **c** CLL B cells + BMSC without contact (separated by a 0.4-μm pore-size filter). Statistical differences were assessed using the paired Wilcoxon non-parametric test

Because microenvironment stimuli are known to influence the cellular physiology of CLL B cells, we measured TET and IDH expression in leukemic cells before and after contact with BMSC (Fig. 3a–e). We observed a downregulation of TET1 (10.31 vs 8.18, *P* = 0.0371) and an upregulation of TET3 (26.73 vs 36.40, *P* = 0.0273) and IDH2 (48.78 vs 84.32, *P* = 0.0039). However, these modifications in mRNA expression were not associated with an alteration in global %5mC (Fig. 3f), as it was similar in both conditions (0.0900 vs 0.0990, *P* = 0.4768). To ensure that BMSC co-culturing did not contaminate the subsequent DNA extraction, we cultured CLL B cells in the conditioned medium of BMSC alone or BMSC + CLL B cells (with and without contact). None of these conditions showed significant differences in terms of %5-hmC as shown in Fig. 4.

### %5-hmC has no prognostic significance in CLL

**Fig. 5:**
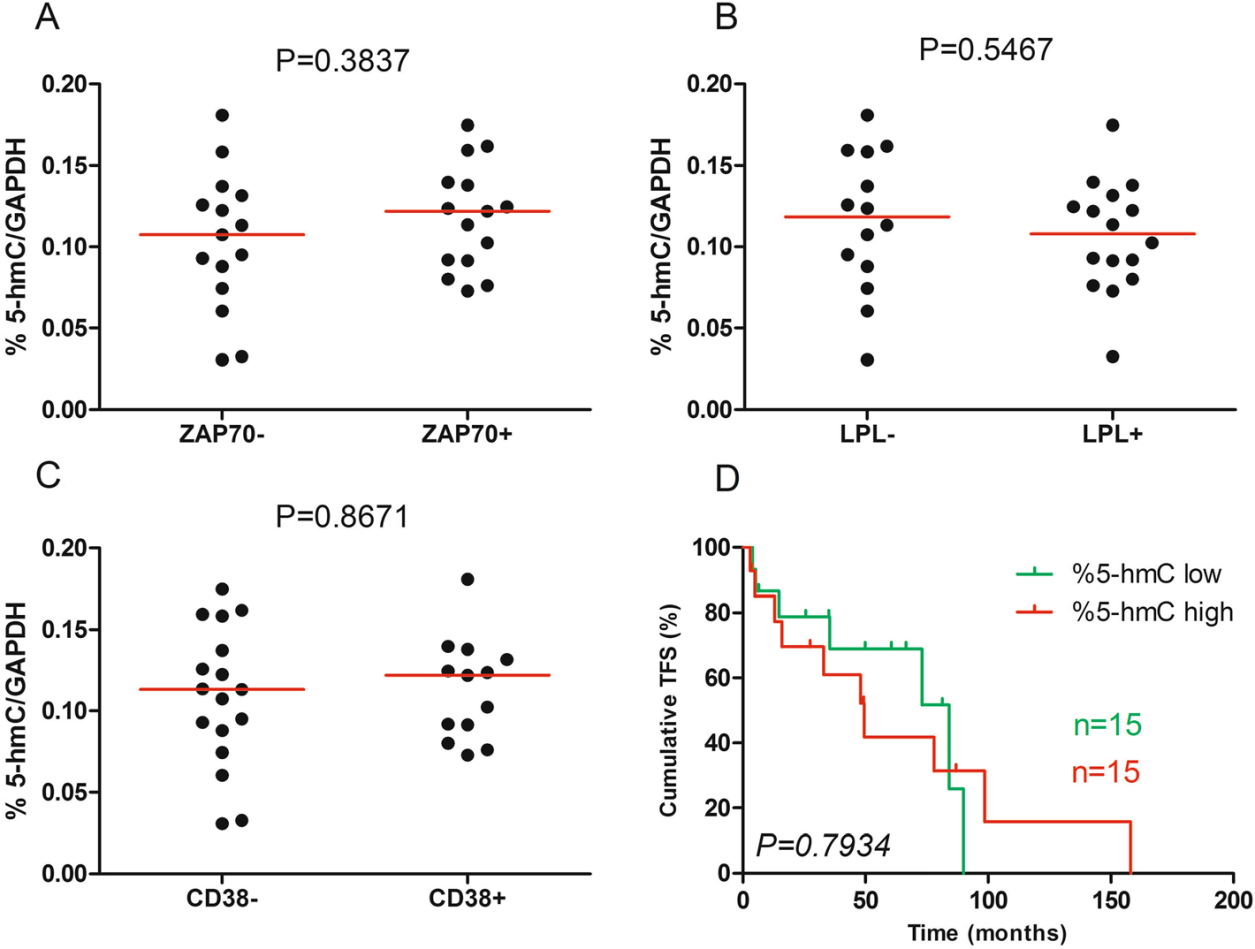
Prognosis significance of 5-hydroxymethylcystosine levels. Normalized %5-hmC was measured by ELISA in B cells from **a** 15 ZAP70− and 15 ZAP70+ patients or **b** 14 LPL− and 16 LPL+ patients or **c** 17 CD38− and 13 CD38+ patients. Median percentages are shown in *red*. Statistical differences were assessed using the Mann–Whitney non-parametric test. **d** Kaplan–Meier curves for TFS are shown for 15 patients with high (*red*) and 15 low (*green*) %5-hmC. The median value was used as the cut-off. Statistical differences between the curves were calculated using the log-rank test

We performed a comparison of %5-hmC between prognostic subgroups based on classical prognostic factors (ZAP70, LPL, and CD38), but we did not observed any significant correlations (*P* = 0.3837, 0.5467, and 0.8671, respectively; Fig. 5a–c). A log-rank test was also performed following Kaplan–Meier analysis of patients displaying low %5-hmC (*n* = 15) vs high %5-hmC (*n* = 15). Using the median as the positive threshold, we did not observe a statistical difference (median TFS of 84 vs 49.57 months, respectively, *P* = 0.7934; Fig. 5d). Other thresholds (based on recursive partitioning or ROC curves maximizing the concordance with the mutational status [14]) gave similar results. OS analysis was not performed due to the absence of deaths among the patients during the study. Similar results were obtained if we normalized %5-hmC to actin or PPIA (peptidyl prolyl isomerase A or cyclophilin A).

### Mutation in TET/IDH genes are rare and non-recurrent in CLL

In order to investigate the mutational profile of TET and IDH genes, we used publically available whole exome and RNAseq data previously published by Quesada et al. [36] and Ferreira et al. [37]. Within the CLL-ES project (accessible on https://dcc.icgc.org/projects/CLLE-ES), simple somatic mutations were analyzed for a total of 218 patients. Six patients (2.8%) presented a mutation for TET1, 5 (2.3%) for TET2, 7 (3.2%) for TET3, 5 (2.3%) for IDH1 and 2 (0.9%) for IDH2 for a total of 25 patients with 27 mutations (Table 1). However, only 3 patients (1.4%) presented a missense mutation respectively in TET1, TET2, and TET3 genes. All the other mutations in these five genes were found in intron, UTR (untranslated region), downstream or upstream the gene indicating that mutations in TET/IDH genes are rare and non-recurrent events compared to what has been observed in other hematological malignancies [27–30].

**Table 1:** TET and IDH gene mutation analysis in 218 CLL patients

Position/mutation	Type	Consequence
TET1		
chr10:g.70318749T>A	Single base substitution	Upstream
chr10:g.70333015C>A	Single base substitution	Missense: P307H
chr10:g.70413592G>A	Single base substitution	Intron
chr10:g.70346080G>A	Single base substitution	Intron
chr10:g.70447656G>A	Single base substitution	Intron
chr10:g.70360941C>G	Single base substitution	Intron
TET2		
chr4:g.106161089G>A	Single base substitution	3 UTR | intron
chr4:g.106196355C>T	Single base substitution	Missense: S1563F, S1584F | 3 UTR
chr4:g.106175952A>C	Single base substitution	Intron
chr4:g.106075153G>A	Single base substitution	Intron
chr4:g.106108570A>T	Single base substitution	Intron
chr4:g.106087334A>C	Single base substitution	Intron
chr4:g.106064903T>A	Single base substitution	Upstream: AC004069.2
TET3		
chr2:g.74276067C>A	Single base substitution	Downstream | intron
chr2:g.74226519->A	Insertion of ≤200 bp	Upstream
chr2:g.74300784G>A	Single base substitution	Exon | intron
chr2:g.74247963G>A	Single base substitution	Intron
chr2:g.74274651G>A	Single base substitution	Missense: R443H, R401H | Exon
chr2:g.74238478A>T	Single base substitution	Intron
chr2:g.74278374G>A	Single base substitution	Downstream
IDH1		
chr2:g.209111428G>A	Single base substitution	Downstream | intron
chr2:g.209123819C>T	Single base substitution	Upstream | downstream IDH1-AS1| intron
chr2:g.209135430A>G	Single base substitution	Upstream-AC016697.2 | intron: PIKFYVE
chr2:g.209121002T>G	Single base substitution	Upstream | downstream: IDH1-AS1 | intron
chr2:g.209102172T>C	Single base substitution	Upstream: AC016697.3 | intron
IDH2		
chr15:g.90631755C>T	Single base substitution	Intron
chr15:g.90631061C>A	Single base substitution	Intron

## Discussion

In the present paper, we performed a comprehensive and complete expression profile of the known proteins involved in DNA hydroxymethylation. To our knowledge, it is the first time that such a study has been performed on a large cohort of CLL patients. In 2013, Hernandez-Sanchez and colleagues observed that TET2 was overexpressed in CLL cells compared with cells from healthy donors [38]. The normal counterpart of CLL cells is not clearly defined in the literature and remains controversial [39]. We therefore decided to use two different controls of normal B cells: first, the peripheral normal B cell from healthy donors since they represent intuitively the normal counterpart in the healthy donors; second, the B cells from the umbilical cord blood since they express the CD5 similarly to CLL [40] and because Saunders et al. demonstrated that they display a similar proteic profile to CLL [41]. In our results, TET2 decreased in CLL cells compared with normal B cells from UCB, but the expression did not differ from that in the PBHV. These discrepancies could be explained by at least three reasons: our cohort was larger than the one used in the previous study (214 vs 23) as well as the control group of PBHV (20 vs 5); the qPCR control in the previous study was GAPDH, while we used PPIA, which is more stable in CLL; and finally, based on the reverse TET2 primer (the forward has been mistaken for the GAPDH reverse primer), their amplicon was in the 3' region (nucleotide 8941 to 8960), while ours was further upstream (nucleotide 4367 to 4389) which is a region less sensible to RNA degradation.

In terms of prognosis, we observed that TET2 and IDH1 were associated with TFS, while the other investigated genes were not; patients expressing a high level of these enzymes had a longer TFS than the low expression group. TET2 is a major factor in hematopoiesis, and several hematological malignancies, such as myelodysplastic syndrome [42], acute myeloid leukemia [43], or chronic myelomonocytic leukemia [44], have been linked to TET2 mutations. IDH1, meanwhile, is a cytosolic enzyme, and the mutated version produces 2-hydroxyglutarate, a truncated form of 2-oxoglutarate that impairs the function of enzymes such as TET [45]. Mutations of TET2 are rare in lymphoid malignancies compared in myeloid malignancies, and based on previous studies, we confirmed in the present work that no relevant mutations in TET or IDH enzymes has been found in CLL cells. However, few mutations of TET2 have been observed in other B cell neoplasms and more in T cell neoplasms [46, 47]. Several studies have also reported a skew toward the myeloid lineage when the TET2 gene is altered [48, 49].

DNA hydroxymethylation, TET enzyme expression, and prognosis have been investigated in other cancers. In acute myeloid leukemia, %5-hmC varied among patients but was correlated with poor OS [50]. In another study, the hypomethylation agent decitabine was used to treat elderly AML patients and increased %5-hmC [51]. TET2 was decreased in cervical squamous cell carcinoma along with %5-hmC, and low levels in patients were shown to be associated with a poor prognosis [52]. In contrast to our results, another team observed that high expression of IDH1 is associated with shorter overall survival in cytogenetically normal acute myeloid leukemia [53]. In epithelial ovarian cancer, TET2 and %5-hmC decreased compared with normal controls, and this low expression was associated with poor overall survival of the patients [54]. The percentage of 5-hmC was decreased in childhood refractory cytopenia as well as TET2 expression. This decreased expression was not associated with the presence of mutations but was correlated with an increase in microRNA-22, which may regulate the enzyme [55]. Another study suggested that loss of %5-hmC may be due to simple nuclear exclusion of the oxidases [56]. For breast cancer, reduced %5-hmC was assessed as a biomarker of tumor development [57], and TET1 decreases were linked to poor prognosis [58]. To our knowledge, no other team has performed a prognostic study of TET enzymes in CLL.

We previously demonstrated that microenvironment stimuli influenced HDAC activity by assessing B cell receptor (BCR) stimulation using anti-IgM antibodies [14]. Here, we observed the effects of BMSC on TET1, TET3, and IDH2 mRNA expression without the global modification of %5-hmC. To confirm that these microenvironment stimuli had no impact on DNA hydroxymethylation, we performed the same analyses on CLL B cells cultured in medium previously conditioned by BMSC or a CLL B cell–BMSC co-culture. Indeed, it is known that cells produce a great variety of soluble factors whose secretion can be modulated in the presence of other cells (with or without contact). Moreover, CLL B cells and BMSC can secrete extracellular vesicles, which act as a means of communication between cells following their integration [59]. Using conditioned medium obtained after centrifugation, we eliminated the possibility of BMSC contamination in the gDNA extraction. As microenvironment stimuli do not affect %5-hmC, it was not surprising that it was similar between different prognostic groups based on classical markers (such as ZAP70, LPL, and CD38 status) because poor disease progression in CLL is often linked to the responsiveness of leukemic cells to their microenvironment. Moreover, %5-hmC was not correlated with clinical data, such as TFS. We hypothesized that, despite TET and IDH enzyme deregulation in CLL, these alterations have no impact on global DNA hydroxymethylation itself. Decrease of TET2 expression by siRNA (data not shown) did not influence the %5-hmC, suggesting that TET2 could have a prognostic impact independently of its enzymatic activity. However, TET enzymes may be involved in other processes, and additional functional studies will be needed to understand their impact on prognosis. TET1, for example, has been identified as a fusion partner in the translocation of the mixed lineage leukemia (MLL) gene in AML [60, 61], and its oncogenic relevance depends more on its capacity to bind DNA and recruit other proteins than its enzymatic activity [15, 62].

## Conclusions

In conclusion, we showed that genes involved in DNA hydroxymethylation are deregulated in CLL and that TET2 and IDH1 are significant predicators of TFS. However, the prognostic value of TET and IDH mRNA deregulation in CLL does not appear to be associated with DNA hydroxymethylation. These data bring biological rationale for further investigations on TET and IDH genes in CLL.

## Additional file

Additional file 1: Text 1, Figure S1, Figure S2, Table S1 Tables S2 and Table S3. (DOCX 1600 kb)

## Abbreviations

5-hmC: 5-Hydroxymethylcytosine
5-mC: 5-Methylcytosine
AML: Acute myeloid leukemia
B2M: Beta-2-microglobulin
BCR: B cell receptor
BMSC: Bone marrow mesenchymal stromal cells
CLL: Chronic lymphocytic leukemia
CMML: Chronic myelomonocytic leukemia
cytog. profile: Cytogenetic profile
GAPDH: Glyceraldehyde-3-phosphate dehydrogenase
HDAC: Histone deacetylase
HRP: Horseradish peroxidase
IDH: Isocitrate dehydrogenases
IgHV: Mutational status of the immunoglobulin heavy-chain variable-region
LDT: Lymphocyte doubling time
LPL: Lipoprotein lipase
OS: Overall survival
PBHV: Peripheral B cells from healthy volunteers
PPIA: Peptidyl prolyl isomerase A or cyclophilin A
qPCR: Quantitative real-time PCR
sCD23: Soluble CD23
SIRT: Sirtuin
TET: Ten-eleven translocation
TFS: Treatment-free survival
UCB: Umbilical cord blood
ZAP70: Zeta-chain-associated protein kinase 70
