# Supplementary material for: Characterization of TET and IDH gene expression in chronic lymphocytic leukemia: comparison with normal B cells and prognostic significance
Source: Clin Epigenetics. 2016 Dec 7;8:132. doi: 10.1186/s13148-016-0298-y (PMC5141649; doi:10.1186/s13148-016-0298-y)
Supplement: Additional file 1: — Text 1, Figure S1, Figure S2, Table S1 Tables S2 and Table S3. (DOCX 1600 kb) [file 13148_2016_298_MOESM1_ESM.docx]

## Additional file 1

## Text 1

### ZAP70, LPL, TET1, TET2, TET3, IDH1 and IDH2 assessment by real-time PCR analysis

We used 25 ng of cDNA (produced by a standard reverse transcription) in a qPCR reaction with SYBR® Green PCR Master Mix (Applied Biosystems) and 0.32 mol/L of gene-specific forward and reverse primers (Invitrogen). We standardized all results using cyclophilin A (PPI A) gene expression. The primer sequences used to amplify ZAP70, LPL, TET1, TET2, TET3, IDH1, IDH2 and PPI are listed in the table below. Standard real-time PCR was performed on an ABI Prism 7900 HT (Applied Biosystems). A calibrator sample (cDNA from the Namalwa cell line, a human B-lymphoid leukemia cell line that expresses ZAP70 at a low level; ATCC) was included as a control in each experiment. In all cases, we created dissociation curves to confirm PCR specificity. Data were analyzed using the comparative ΔΔCt method.

| **Symbol** | **Gene description** | **Forward primer** | **Reverse primer** |
| --- | --- | --- | --- |
| ZAP70 | Zeta-associated protein 70 | GTTGACTCATCCTCAGAGACGAAT | AGGTTATCGCGCTTCAGGAA |
| LPL | Lipoprotein lipase | CCGCCGACCAAAGAAGAGAT | TTCCTGTTACCGTCCAGCCAT |
| TET1 | Ten-eleven translocation methylcytosine dioxygenase 1 | CACATAAGATAAGGGCAGTGGAAA | TTCAGGTTGCACGGTCTCAGT |
| TET2 | Ten-eleven translocation methylcytosine dioxygenase 2 | GGATGACCCAAAAGAGGAAGAGA | GTGCCATAAGAGTGGACAGGTTT |
| TET3 | Ten-eleven translocation methylcytosine dioxygenase 3 | GGACCTGGCCACCGAAGT | GACGGCAGTCAATCGCTATTTC |
| IDH1 | Isocitrate dehydrogenase 1 | CGGAACCCAAAAGGTGACAT | TGGCAACACCACCACCTTCT |
| IDH2 | Isocitrate dehydrogenase 2 | CACGGCCTCAGCAATGTG | TCGAGGAAGTCCGTGGTGTT |
| PPI A | Cyclophilin A | GCTCGTGCCGTTTTGCA | GCAAACAGCTCAAAGGAGACG |

### Primer sequences of genes used for 5-hmC level normalization

| **Symbol** | **Gene description** | **Forward primer** | **Reverse primer** |
| --- | --- | --- | --- |
| GAPDH | Glyceraldehyde-3-phosphate dehydrogenase | CAAGGCTGTGGGCAAGGT | GGCCATGCCAGTGAGCTT |
| Actin | Actin | GCGCGGCTACAGCTTCA | CTTAATGTCACGCACGATTTCC |
| PPI A | Cyclophilin A | GCTTTGGGTCCAGGAATGG | GTTGTCCACAGTCAGCAATGGT |

### CD38 assessment by flow cytometry (FC)

We evaluated the cell surface expression of CD38 by FC in a CD19+ gate with a panel of fluorochrome-labeled monoclonal antibodies (phycoerythrin-conjugated CD38, cyanine-5 CD19, Immunotech). CD38 expression was deemed positive if 7% of the cells stained positive in a standard 3-color FC analysis. This cut-off was calculated using ROC curve analysis, maximizing the concordance with IgHV mutational status [1].

### sCD23 and beta-2-microglobulin by ELISA immunoassay

sCD23 and beta-2-microglobulin serum levels were determined using commercial immunoassay kits (Life Technologies, Gent, Belgium and RayBiotech, Norcross, USA). Standards were used to fully quantify the sCD23 or β2-microglobulin level, and the provided controls were assessed in each experiment to monitor the assay performance and the inter-assay variability.

### Lymphocyte doubling time assessment

Lymphocyte doubling time was determined as described by Montserrat et al. [2] and is defined as the time needed to double the peripheral lymphocyte count.

### Cytogenetic abnormality assessment

For conventional cytogenetic analysis, culture conditions, harvesting, slide preparation, and G-banding were carried out as described previously [3]. Additional cytogenetic abnormalities were investigated with the Chromoprobe Multiprobe® CLL System. Fresh or frozen CLL cells were washed twice with PBS and incubated in KCl (0.075 M, pH7) for 10 min. Cells were then fixed with Carnoy’s fixative (3:1 methanol: glacial acetic acid). Hybridization was performed according to the manufacturer’s recommendation. The cells (100 to 200) were counted to generate representative results. The CLL FISH panel allows for the detection of trisomy 12, deletions in 13q14 ATM (11q22.3), TP53 (17p13.1) and MYB (6q23.3) and also translocations involving IGH Fission (14q32), IGH/CCND1 (14q32/11q13.3) and IGH/BCL2 (14q32/18q21.3).

### IgHV gene mutational analysis

IgHV gene mutational analysis was performed with an IGH Somatic Hypermutation Assay v2.0 (Invivoscribe; La Ciotat, France), and sequences were aligned with those in the international ImMunoGeneTics information system database (http://imgt.cines.fr). Sequences with $\leq$ 2% deviation from any germ line IgHV sequence were considered unmutated [4].

## References

(1) Stamatopoulos B, Meuleman N, Haibe-Kains B, Duvillier H, Massy M, Martiat P, et al. Quantification of ZAP70 mRNA in B cells by real-time PCR is a powerful prognostic factor in chronic lymphocytic leukemia. Clin Chem 2007 Oct;53(10):1757-66.

(2) Montserrat E, Sanchez-Bisono J, Vinolas N, Rozman C. Lymphocyte doubling time in chronic lymphocytic leukaemia: analysis of its prognostic significance. Br J Haematol 1986 Mar;62(3):567-75.

(3) Heimann P, Devalck C, Debusscher C, Sariban E, Vamos E. Alveolar soft-part sarcoma: further evidence by FISH for the involvement of chromosome band 17q25. Genes Chromosomes Cancer 1998 Oct;23(2):194-7.

(4) Hamblin TJ, Davis Z, Gardiner A, Oscier DG, Stevenson FK. Unmutated Ig V(H) genes are associated with a more aggressive form of chronic lymphocytic leukemia. Blood 1999 Sep 15;94(6):1848-54.

## additional Figure Legend

**Figure S1. Prognostic power of classical prognostic factors.** (A and J), TFS and OS curves for Binet stage A vs B-C (n=214); (B and K) IgHV mutational status (n=208); (C and L), LDT (n=180); (D and M), ZAP70 by qPCR (n=214); (E and N), LPL by qPCR (n=214); (F and O), CD38 (n=214); (G and P), cytogenetic abnormalities detected by classical karyotype analysis or by FISH (normal/del(13q)/other) vs (del(17p)/(11q)/(6q)/+12/complex) (n=159); (H and Q), sCD23 (n=175) ; (I and R), B2M (n=187). ROC curves were used to determine the ZAP70, LPL, CD38, sCD23 and B2M expression cut-off values that best distinguished mutated and unmutated cases. IgHV mutational status is based on a 98% cut-off value. Statistical differences between curves were calculated using the log-rank test. Statistical details can be found in Table S1.

**Figure S2. ΔCt representation of TET and IDH in CLL and control samples.** ΔCt of (A) TET1, (B) TET2, (C) TET3, (D) IDH1, and (E) IDH2 in CD19 purified cells from 214 CLL, 20 peripheral blood of healthy patients (PBHV) and 21 umbilical cord blood (UCB) samples are displayed with median. Statistical differences are indicated in relation to CLL and were assessed using the Mann-Whitney non-parametric test.


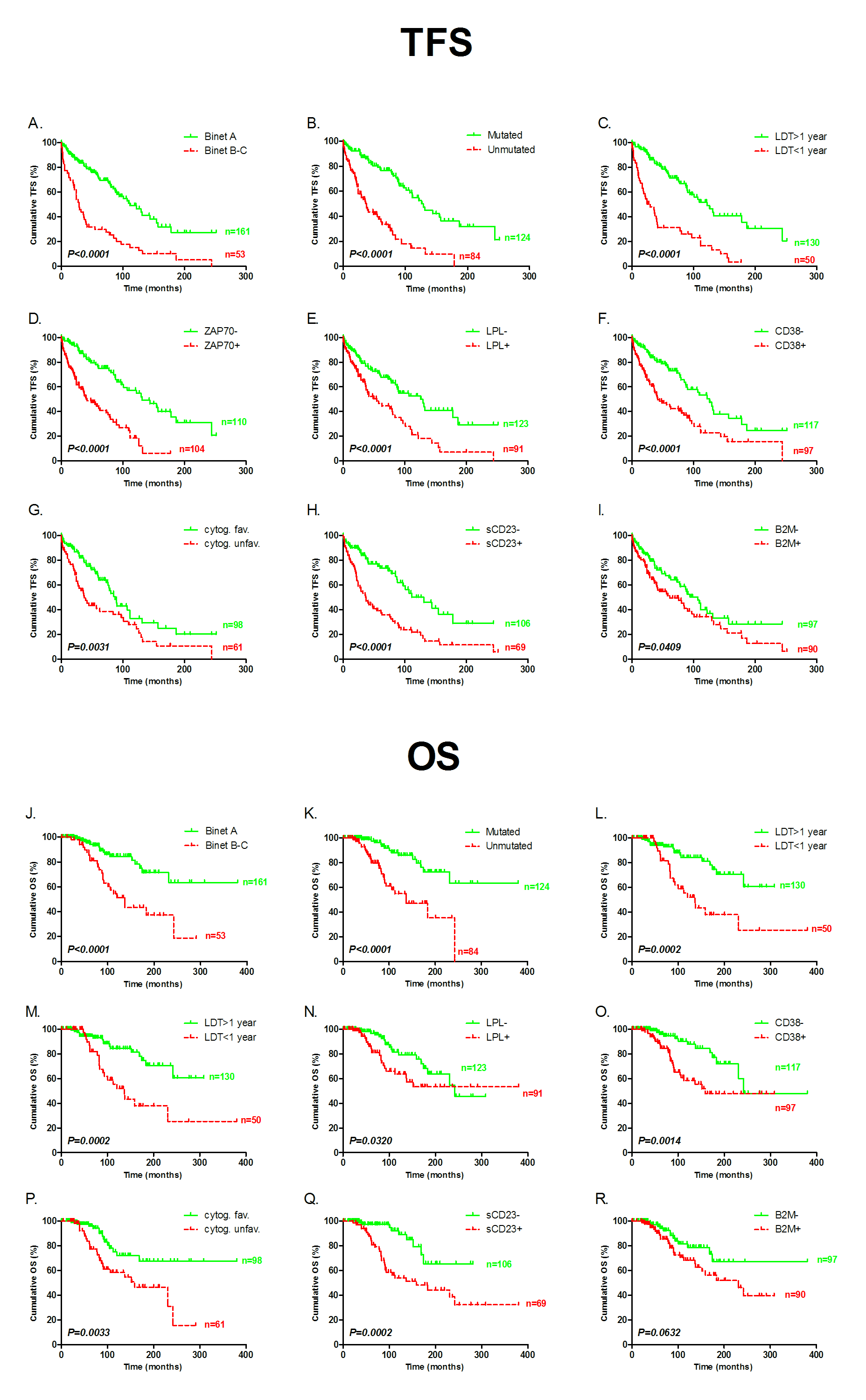


Figure S1. Prognostic power of classical prognostic factors.


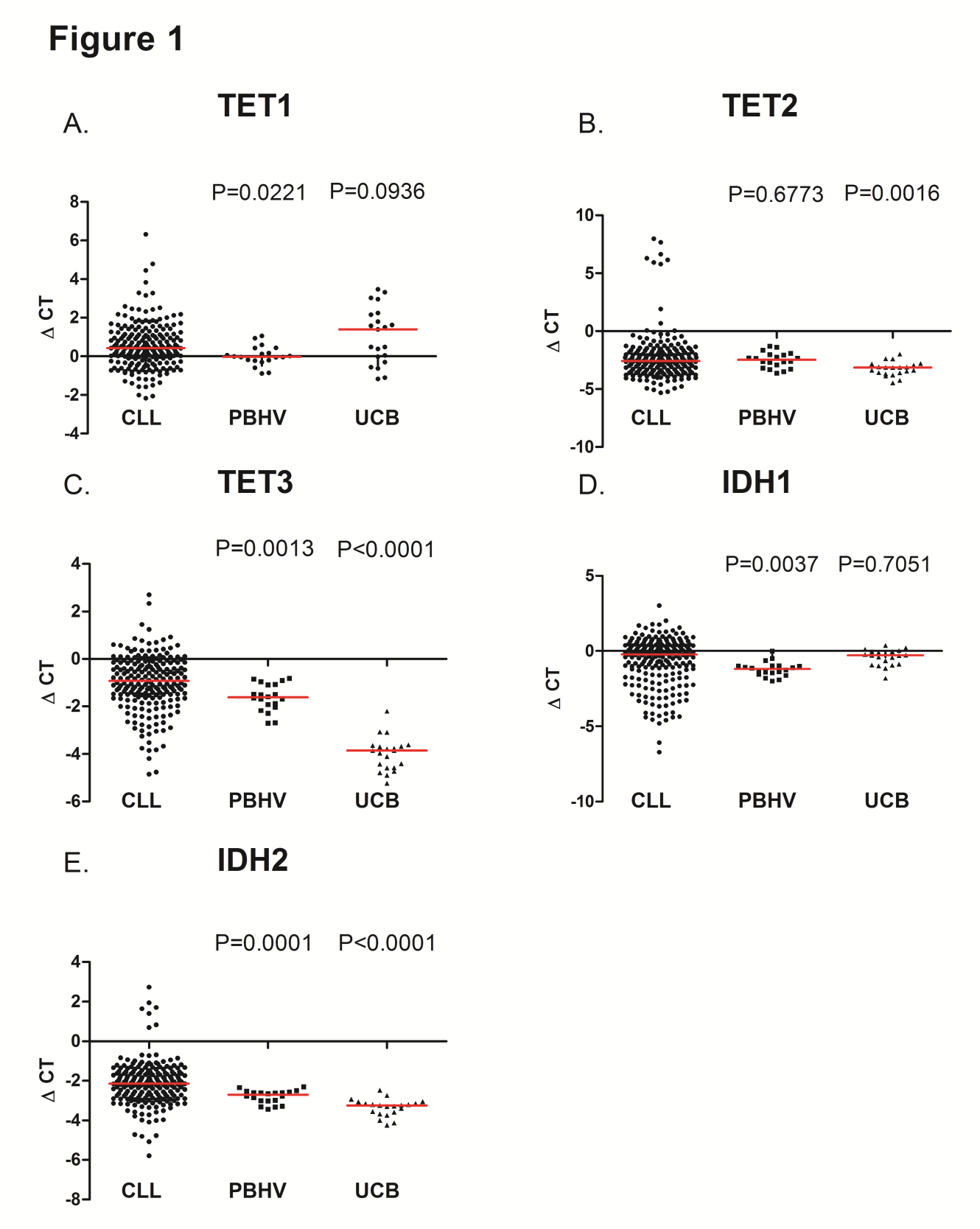


**Figure S2. ΔCt representation of TET and IDH in CLL and control samples.**

Table S1. Prognostic power of classical prognostic factors

Table S2. Patients characteristics and TET gene expression in different prognostic groups

Table S3.Patients characteristics and IDH gene expression in different prognostic groups
